# Nanopublications for exposing experimental data in the life-sciences: a Huntington’s Disease case study

**DOI:** 10.1186/2041-1480-6-5

**Published:** 2015-02-09

**Authors:** Eleni Mina, Mark Thompson, Rajaram Kaliyaperumal, Jun Zhao, van Eelke der Horst, Zuotian Tatum, Kristina M Hettne, Erik A Schultes, Barend Mons, Marco Roos

**Affiliations:** Department of Human Genetics, Leiden University Medical Center, PO Box 9600, 2300 RC Leiden, The Netherlands; Department of Zoology, University of Oxford, Oxford, UK

**Keywords:** Huntington’s disease, Nanopublication, Provenance, Research object, Workflows, Interoperability, Data integration

## Abstract

Data from high throughput experiments often produce far more results than can ever appear in the main text or tables of a single research article. In these cases, the majority of new associations are often archived either as supplemental information in an arbitrary format or in publisher-independent databases that can be difficult to find. These data are not only lost from scientific discourse, but are also elusive to automated search, retrieval and processing. Here, we use the nanopublication model to make scientific assertions that were concluded from a workflow analysis of Huntington’s Disease data machine-readable, interoperable, and citable. We followed the nanopublication guidelines to semantically model our assertions as well as their provenance metadata and authorship. We demonstrate interoperability by linking nanopublication provenance to the Research Object model. These results indicate that nanopublications can provide an incentive for researchers to expose data that is interoperable and machine-readable for future use and preservation for which they can get credits for their effort. Nanopublications can have a leading role into hypotheses generation offering opportunities to produce large-scale data integration.

**Electronic supplementary material:**

The online version of this article (doi:10.1186/2041-1480-6-5) contains supplementary material, which is available to authorized users.

## Background

The large amount of scientific literature in the field of biomedical sciences makes it impossible to manually access and extract all relevant information for a particular study. This problem is mitigated somewhat by text mining techniques on scientific literature and the availability of public online databases containing (supplemental) data. However, many problems remain with respect to the availability, persistence and interpretation of the essential knowledge and data of a study.

Text mining techniques allow scientists to mine relations from vast amounts of abstracts and extract explicitly defined information [1] or even implicit information [2, 3]. Because most of these techniques are limited to mining abstracts, it is reasonable to assume that information such as tables, figures and supplementary information are overlooked. Moreover, recent attempts to mine literature for mutations stored in databases, showed that there was a very low coverage of mutations described in full text and supplemental information [4].

This is partly remedied by making data public via online databases. However, this by itself does not guarantee that data can be readily found, understood and used in computational experiments. This is particularly problematic at a time when more, and larger, datasets are produced that will never be fully published in traditional journals. Moreover, there is no well-defined standard for scientists to get credit for the curation effort that is typically required to make a discovery and its supporting experimental data available in an online database. We argue that attribution and provenance are important to ensure trust in the findings and interpretations that scientists make public. Additionally, a sufficiently detailed level of attribution provides an incentive for scientists, curators and technicians to make experimental data available in an interoperable and re-usable way. The Nanopublication data model [5] was proposed to take all these issues into consideration. The nanopublication guidelines document [5] provides details of the nanopublication schema and recommendations for constructing nanopublications from Life Science data. Based on Semantic-web technology, the nanopublication model is a minimal model for publishing an assertion, together with attribution and provenance metadata.

The assertion graph contains the central statement that the author considers valuable (publishable) and for which she would like to be cited (attribution). It should be kept as small as possible in accordance with the guidelines. The provenance graph is used to provide evidence for the assertion. It is up to the author to decide how much provenance information to give, but in general, more provenance will increase the trustworthiness of the assertion, and thus the value of the nanopublication. The publication info graph provides detailed information about the nanopublication itself: creation date, licenses, authors and other contributors can be listed there. Attribution to curators and data modelers are part of the nanopublication design to incentivize data publishing.

We used the nanopublication schema to model scientific results from an in-silico experiment. Previously Beck *et al.* [6] used GWAS data stored in the GWAS central database to model as nanopublications and they demonstrated how such valuable information can be incorporated within the Linked Data web to assist the formation of new hypotheses and interesting findings. In our experiment we investigated the relation between gene deregulation in Huntington’s disease and epigenetic features that might be associated with transcriptional abnormalities (E. Mina *et al.*, manuscript in preparation).

We show how the results of this case study can be represented as nanopublications and how this promotes data integration and interoperability.

### Huntington’s Disease as case study for modelling scientific results into nanopublications

Huntington’s Disease is a dominantly inherited neurodegenerative disease that affects 1 - 10/100.000 individuals and thus making it the most common inherited neurodegenerative disorder [7]. Despite the fact that the genetic cause for HD was already identified in 1993, no cure has yet been found and the exact mechanisms that lead to the HD phenotype are still not well known. Gene expression studies revealed massive changes in HD brain that take place even before first symptoms arise [8]. There is evidence for altered chromatin conformation in HD [9] that might explain these changes. We selected to analyse two datasets that are associated with epigenetic regulation, concerning CpG islands in the human genome [10] and chromatin marks mapped across nine cell types [11]. Identifying genes that are deregulated in HD and are associated with these regions can give insight into chromatin-associated mechanisms that are potentially at play in this disease.

Our analysis has been implemented through the use of workflows using the Taverna workflow management system [12, 13]. As input we used gene expression data from three different brain regions from HD affected individuals and age and sex matched controls [14]. We tested for gene differential expression (DE) between controls and HD samples in the most highly affected brain region, caudate nucleus, and we integrated this data with the two epigenetic datasets discussed previously which are publicly available via the genome browser [15, 16].

HD is a devastating disease and no actual cure has been found yet to treat or slow down disease progression. Therefore, research on this domain is mainly focusing on the production of new data and investment on expensive experiments. It is important to realize that sharing information is essential in research for developing new hypotheses that can tackle difficult use cases such as HD. Because of the unavailability of previous experiments to be found online using common biomedical engines, expensive experiments become lost and unnecessarily replicated. For example in our case study, we found that the association that we inferred between the HTT gene, which mutant form causes Huntington’s Disease, and BAIAP2, a brain-specific angiogenesis inhibitor (BAI1)-binding protein, was present in a table in a paper by Kaltenbach *et al.* [17]. However, it is not explicitly in any abstract which makes it hard to retrieve from systems such as PubMed.

## Results and Discussion

### Nanopublication model design principles

We decided to model and expose as nanopublications two assertions from the results of our workflow: 1) differentially expressed genes in HD and 2) genes that overlap with a particular genomic region that is associated with epigenetic regulation. Note that these natural language statements would typically be used in a caption for a figure, table or supplemental information section to describe a dataset in a traditional publication. Considering the problems with automatic retrieval and interpretation of such data, we aim to expose these assertions in a way that is more useful to other scientists (for example to integrate our results with their own data). Moreover, we provide provenance containing the origin and experimental context for the data in order to increase trust and confidence. Our nanopublications are stored in the AllegroGraph triple store [18]. The link to the browsable user interface and the SPARQL endpoint can be found on the myExperiment link: http://www.myexperiment.org/packs/622.html. The user can log in and browse through the nanopublications by logging in with username “test” and password “tester”. The queries used in this paper are stored under the menu “Queries → Saved”.

#### Assertion model

We defined two natural language statements that we wish to convert to RDF:

“gene X is associated with HD, because it was found to be deregulated in HD” and “gene Y is associated with a promoter, and this promoter overlaps with a CpG island and/or a particular chromatin state”, and we wish to refer to the experiment by which we found these associations. We decided to model our results into two nanopublications. By further subdividing those statements, we see the RDF triple relations appear naturally:

##### Nanopublication assertion 1:

1. There is a gene disease association that *refers_to* gene X and Huntington’s Disease

##### Nanopublication assertion 2:

**Figure 1:**
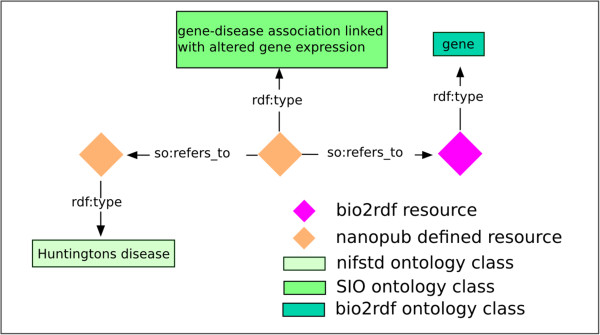
The template for the differential gene expression assertion. Orange diamonds refer to a RDF resource that was defined by this nanopublication, whereas the gene (pink diamond) is defined by a bio2rdf resource. The Sequence Ontology (SO) were used for the predicates refers_to. The classes for Huntington’s disease, gene and gene-disease association linked with altered gene expression are defined by the nifstd, bio2rdf and SIO ontologies respectively

**Figure 2:**
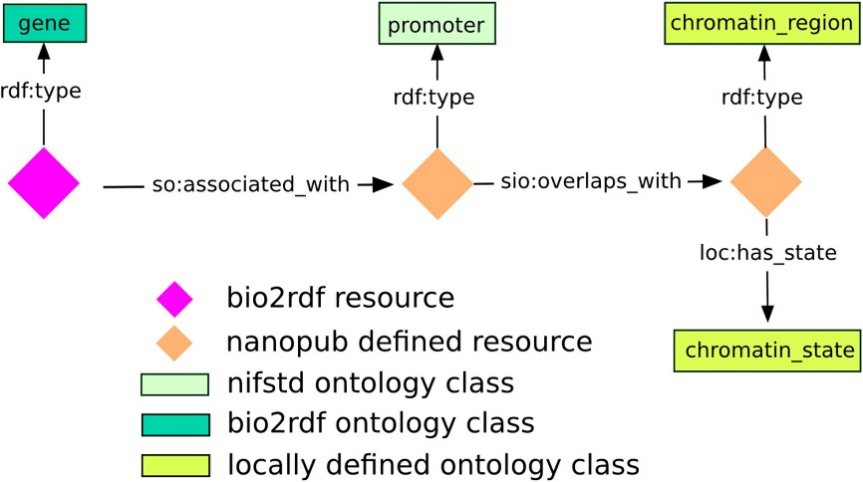
Orange diamonds refer also here to a RDF resource that was defined by this nanopublication, whereas the gene (pink diamond) is defined by a bio2rdf resource. The classes promoter and biological region were defined by the nifstd ontology. The Semanticscience Integrated Ontology (SIO) and Sequence Ontology (SO) were used for the predicates overlaps_with and associated_with

1. Gene Y is *associated_with* promoter Z
2. Promoter Z *overlaps_with* a biological region^a^The assertion models are shown in Figure 1 and Figure 2.

For some of the terms in these statements we found several ontologies that defined classes for them. For example, “promoter”, “gene”, and “CpG island” appear (among others) in the following ontologies: NIF Standard ontology (NIFSTD), NCI Thesaurus (NCI) and the Gene Regulation Ontology (GRO)^b^. We chose to use NIFSTD for our case study, because it covers an appropriate domain and it uses the Basic Formal Ontology (BFO), which can benefit data interoperability and OWL reasoning (e.g. for checking inconsistencies).

**Figure 3:**
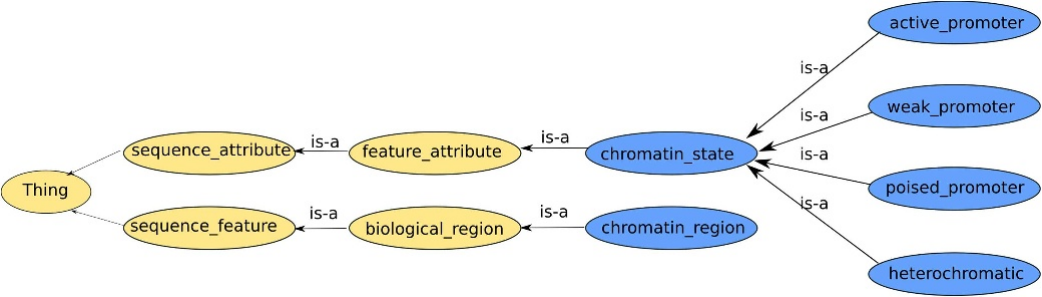
Schematic representation of the extension of SO with our own defined classes. In yellow are depicted the SO ontology classes and in blue the classes we defined in our case study

We chose to use bio2rdf instances for the associated genes [19] because they provide RDF with resolvable resource URIs for many different biomedical resources. To describe the gene-disease association linked with altered gene expression we used the class with that label from the SemanticScience Integrated Ontology (SIO) [20]. The SIO predicate “refers to” was used to associate each differentially expressed gene with HD. There were also terms that we did not find in an available ontology. These were the ones that described the type of the chromatin state that a promoter of a gene can be in, “active promoter state”, “weak promoter state”, “poised promoter state” and “heterochromatic”. We decided to create our own classes to describe these terms. Being aware of interoperability issues, we defined them as subtypes of classes in the Sequence Ontology (SO). We defined the class “chromatin_region” as a subclass of “biological_region” in SO. We defined another class “chromatin_state” as a subclass of “feature_attribute”. Subclasses of “chromatin_state” are the states “active_promoter”, “weak_promoter”, “poised_ promoter” and “heterochromatic”, Figure 3. In Table 1 we provide the definition for these classes, the reused SO ontological terms and their corresponding URIs. We also defined an object property “has_state” which has domain chromatin and range “chromatin_state”.

**Table 1:** **Definition of new classes**

Class	URI	Definition	subclass of	subclass of URI
chromatin_region	biosemantics:chromatin_region	A region of chromatin, likely to	biological_region	so:SO_0001411
		be involved in a biological process		
chromatin_state	biosemantics:chromantin_state	Annotation of chromatin states, defined by	feature_attribute	so:SO_0000733
		combinations of chromatin modification patterns		
		(described in publication by Ernst et al. Nature, 2011)		
active_promoter	biosemantics:active_promoter	Open chromatin region, associated with promoters,	chromatin_state	biosemantics:chromatin_state
		transcriptionally active, defined by the most highly		
		observed chromatin marks : H3K4me2,H3K4me3, H3K27ac,		
		H3K9ac		
weak_promoter	biosemantics:weak_promoter	Open chromatin region, associated with promoters,	chromatin_state	biosemantics:chromatin_state
		weak transcription activity, defined by the most highly		
		observed chromatin marks : H3K4me1, H3K4me2,H3K4me3,		
		H3K9ac		
poised_promoter	biosemantics:poised_promoter	Open chromatin region, associated with promoters,	chromatin_state	biosemantics:chromatin_state
		described as a bivalent domain that has strong signals		
		of both active and inactive histone marks. Most highly		
		observed histone marks: H3K27me3, H3K4me2, H3K4me3		
heterochromatic	biosemantics:heterochromatic	Closed chromatin formation, transcriptionally inactive.	chromatin_state	biosemantics:chromatin_state
		It is associated with none histone marks		

Prefix biosemantics: http://rdf.biosemantics.org/ontologies/chromatin#.

Prefix so: http://purl.obolibrary.org/obo/.

For the predicates we considered the use of the Relation Ontology. Extending the Relation Ontology with the appropriate predicates would support interoperability and reasoning in the long term, because its use of BFO. However, we found that the OWL domain and range specifications did not match our statements. Therefore, we decided to use predicates from the also popular Sequence Ontology (SO) and Semanticscience Integrated Ontology (SIO) [20] that also seemed appropriate for our assertions. This is a typical trade-off between quality and effort that we expect nanopublishers will have to make frequently. We can justify this for two reasons: 1) releasing experimental data as linked open data using any standard ontology is already an important step forward from current practice and 2) interoperability issues at the ontology level is a shared responsibility with ontology developers and curators who provide mappings between ontologies and with higher level ontologies.

The process of nanopublication modeling can be minimized when previous examples are used as *templates* for similar data. For instance, the nanopublication models presented here can serve as templates for exposing differentially expressed genes in a disease condition. We demonstrate the reuse of our own template of Figure 2 by exposing 5 types of nanopublications concerning genomic overlap. The reuse of templates improves interoperability of scientific results beyond the interoperability that RDF already provides. It facilitates crafting assertions while ensuring that the same URIs are used for the same type of data.

#### Provenance model

Publishing information is meaningful only if there is enough supporting information for reproducing them. For example Ioannidis *et al.*, pointed out that they could not reproduce the majority of the 18 articles they investigated describing results from microarray experiments, including selected tables and figures [21]. Nanopublication does not guarantee full reproducibility, but as a model for combining data with attribution and provenance in a digital format it at least makes it possible to trace the origin of scientific results. The provenance section of a nanopublication ties the results (the nanopub assertion) to a description of an experiment and the associated materials, conditions and methods. The main purpose is to capture as accurately as possible where the assertion came from and what the conditions of our experiment were by aggregating and annotating resources that were used throughout the experiment.

In our case the experiment is in-silico: a workflow process that combines existing data sources to expose new associations. Details and references to the original datasets, the workflow process itself and the final workflow output are interesting provenance as they increase trust in the assertion and make it possible to trace back the results of the experiment. An extra benefit of using workflows is that provenance information can be automatically generated by the workflow system and additional tools can be used to associate a workflow with additional metadata and resources. We used Taverna to build and execute our workflows [12]. Taverna provides an option to export the provenance of a workflow execution in prov-o [22].

**Extending nanopublication provenance with the Research Object model**

With the nanopublication provenance model as a starting point we further enhance provenance with a model that has been developed for bundling workflows with additional resources in the form of workflow-centric Research Objects.

Additional resources may include documents, input and output data, annotations, provenance traces of past executions of the workflow, and so on. Research objects enable in silico experiments to be preserved, such that peers can evaluate the method that led to certain results, and a method can be more easily reproduced and reused. Similar to nanopublications, the Research object model is grounded in Semantic Web technologies [23]. It is comprised by a core ontology and extension ontologies. The core ontology reuses the Annotation Ontology (AO) and the Object Reuse and Exchange (ORE) model to provide annotation and aggregation of the resources. The extension ontologies keep track of the results and methods of a workflow experiment (wfprov), provide the descriptions of scientific workflows (wfdesc) and capture the RO evolution process (roevo) (Belhajjame K, Zhao J, Carijo D, Hettne KM, Palma R, Mina E, Corcho O, Gómez-Pérez JM, Bechhofer S, Klyne G, Goble C: Using a suite of ontologies for preserving workflow-centric research objects, accepted for publication Journal of Web Semantics). Research objects extend the already existing functionality of my Experiment packs. We created Research objects using the Research object repository sandbox, which offers a user friendly interface for creating Research objects either by importing an already existing pack from my Experiment, or uploading a.zip archive or creating a research object manually [24].

**Figure 4:**
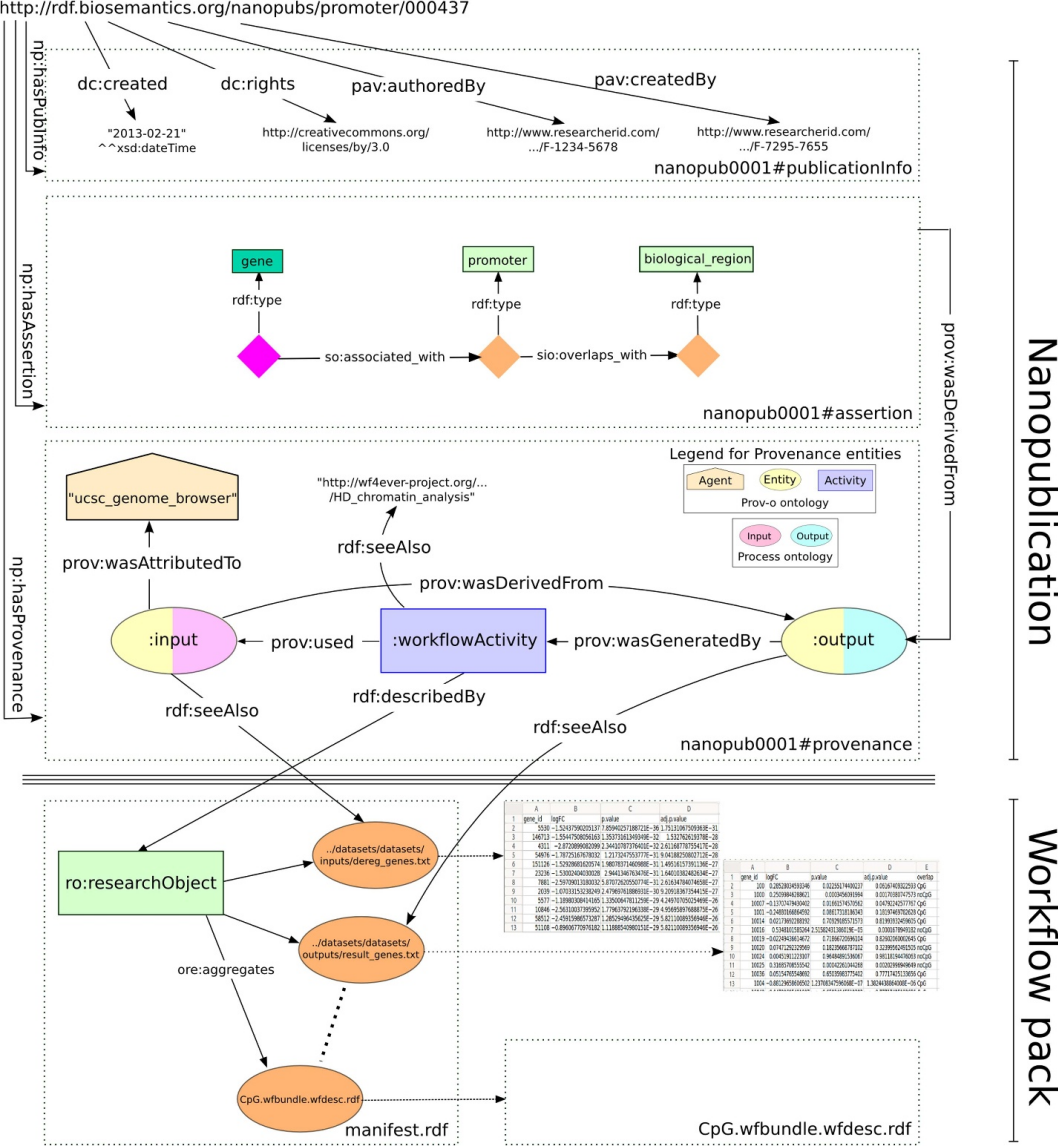
The top part of this figure (above the triple line) shows the Nanopublication consisting of its three constitutive parts: Assertion, Provenance and Publication Info. Below the triple line is the Workflow pack which contains the Research Object (RO) as well as all the input and output data, workflows and results for the experiment. Note that the nanopublication and the workflow pack are separate entities that can exist in different locations. The two models are linked by the predicates shown in the figure as arrows crossing the triple line. In this way the nanopublication re-uses and exposes the detailed (and partially automatically generated) provenance from the RO. Note that multiple nanopublications can reference the same RO. Not shown in the figure is the possibility for the RO to link back to the nanopublication as one of the results of the experiment described by the RO

An overview of the connection between the Nanopublication model and the Research Object is given in Figure 4. In the nanopublication provenance graph we include a simple provenance model that describes the context of the workflow process: in particular the relation of the nanopublication assertions as the origin of the experiment outputs. Note that the workflow activity links to the Research Object and each of the input/output entities link to the corresponding entity in the Research Object. This way, the nanopublication provenance serves as a proxy for the Research Object, such that larger nanopublication collections can be queried without downloading all Research Objects. Moreover, we increase interoperability by using the standard Prov-o ontology in the nanopublication provenance, to which the Research Object ontology is aligned. Futhermore we increase the semantics of the input/output entities by using the domain specific Process ontology [25]. In summary, the strength of linking the entities in a nanopublication provenance to a Research Object, is to augment the experimental context information which is key evidence for the statement made in the assertion. Moreover, this link enables reuse of provenance information that may be already available. However, we note that deciding the amount and relevance of provenance information to be included in the nano-publication remains a decision of the nanopublication author.

#### Publication info

In the *Publication Info* section of a nanopublication we capture details that are required for citation and usage of the nanopublication itself. The authors of the nanopublication and possible contributors are described here, and represented by a unique research identifier to account for author ambiguity. The timestamp of the nanopublications creation is also recorded in this part, as well as versioning details. Finally, information about usage rights and licence holders is included.

### Data integration using nanopublications: assisting drug target prioritization in HD

By choosing RDF as exchange format for nanopublications, we also support the data integration features of RDF. In HD research, diverse working groups recruit a variety of disciplines that produce data encompassing brain images, gene expression profiles in brain and peripheral tissues, genetic variation, epigenome data, etcetera, with the common goal to identify biomarkers to monitor disease progression or the effectiveness of therapies. Nanopublications provide an incentive to expose this data such that we can more easily integrate them with each other to assist research in HD by creating novel hypotheses. These hypotheses can be further tested and ultimately help the development of effective treatments. Following standardized templates to model information ensures data interoperability that can facilitate complex queries for discovering new information. In addition, the attached provenance of the assertion will give necessary information related to the experiment, to ensure trust but also to be able to reuse the scientific protocol and replicate the results. Moreover, we note that nanopublications enable opportunities for data integration beyond the assertions and the experimental data itself. Since nanopublication *Provenance* relates to the methodology/protocol that was used as part of an experiment, it allows us to retrieve all other published nanopublications based on our workflow, or workflows that are related to it (e.g. because they use the same kind of input data). Such (indirect) provenance links greatly improve the discoverability of research data. Another option could be to use the information stored in the Publication Info graph and retrieve the attribution information for the nanopublication. This makes it relatively easy to determine the most frequently cited nanopublication creators and authors, for example in order to calculate some kind of impact factor.

To demonstrate how data integration with nanopublications can occur in practice we applied simple SPARQL queries to our local nanopublication store. For example, to identify differentially expressed genes for which the promoters are associated both with a CpG island and a poised chromatin state. The resulted genes can be the start for further research, as they may be indicators for an epigenetically mediated gene alteration. The set of the canned queries are stored in our nanopublication store for the user to browse and execute (details for accessing the nanopublication store were mentioned previously in this paper)

Nanopublications can also facilitate more sophisticated queries to support data integration. Note that traditional methods of querying integrated data would typically require converting data sources to a common format or database and writing one or more queries and scripts to solve the actual question. Such an approach can be complex and time-consuming, while the resulting code and data is not necessarily re-usable to answer other research questions. Here we show a complex data integration question that can be answered using a relatively short SPARQL query.

**Figure 5:**
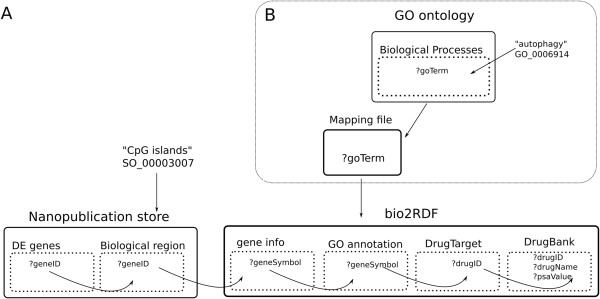
First we queried the differentially expressed genes nanopublication to get all gene ids. Further we filtered these ids by querying the biological region nanopublication. Subsequently we queried the bio2RDF GO annotation dataset to select genes that are involved in the biological processes mentioned in the text. For this gene list we queried bio2RDF gene information data source to retrieve gene symbols that belong to the human taxonomy, that were used to retrieve the drug target from bio2RDF drugbank. Last, these drug targets are used to find drugs from the bio2RDF drugbank source. **B**. The process of querying GO for our set of biological processes(including the children concepts) and the mapping URI to filter genes from the bio2RDF GO annotation dataset. In this example we retrieve drug targets for one of the GO terms, autophagy.

**Figure 6:**
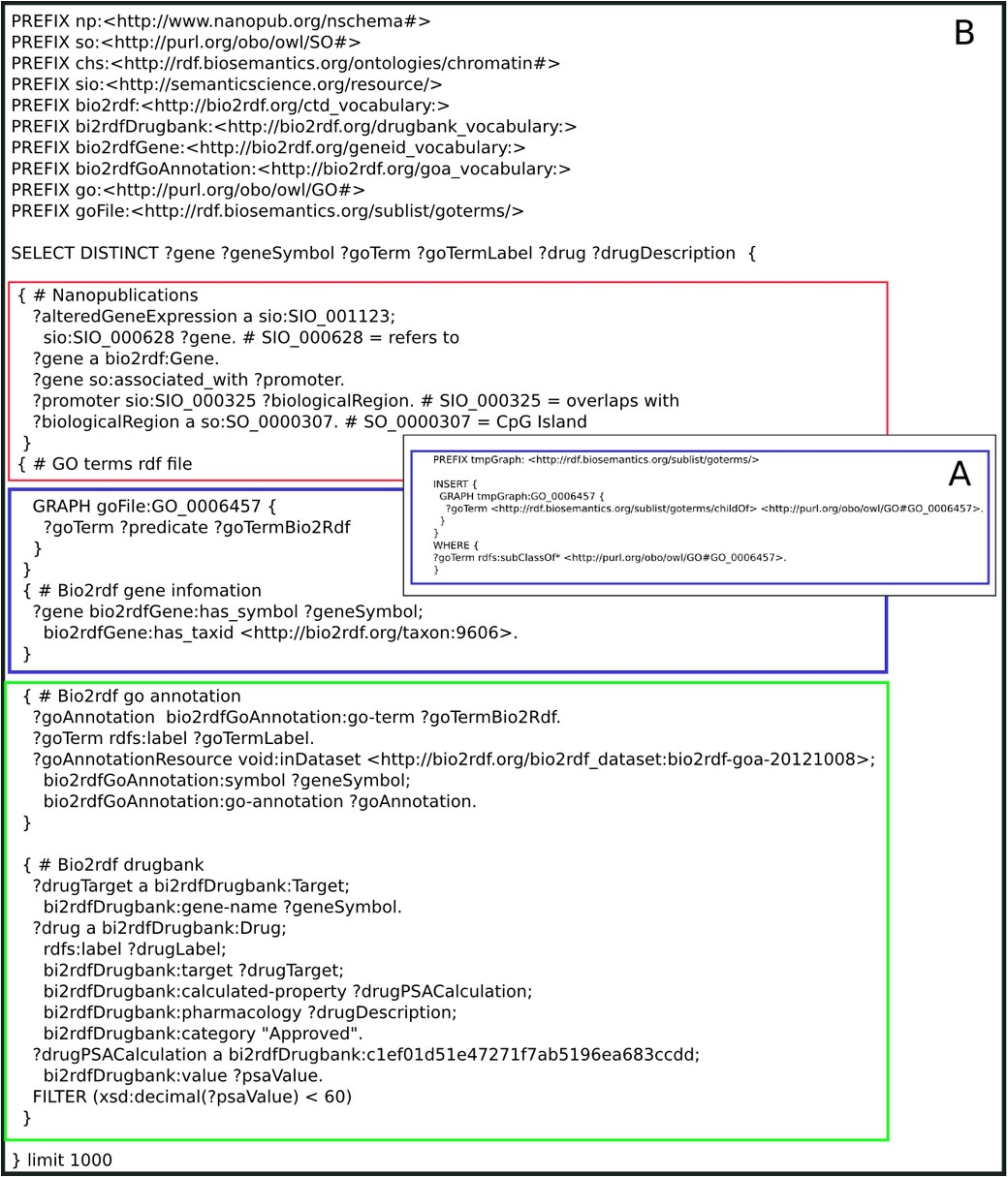
SPARQL query for retrieving and adding GO terms from the GO ontology to our local graph. **B**. The example SPARQL query that retrieves drug targets and drug names from Drugbank that are associated with the genes that we identified as differentially expressed in Huntington’s Disease and overlap with CpG islands. red: our local nanopublication store; blue: the mapping URI; green: bio2RDF data sources.

In this case we would like to identify druggable targets that target four biological processes that (among others) are impaired in HD [26, 27]. These were the proteasomal protein catabolic process (GO:0010498), autophagy (GO:0006914), protein folding (GO:0006457) and protein unfolding (GO:0043335). We identified drug targets by querying across four different data sources, including drugbank and pathway data, as schematically shown in Figure 5. These targets can be a valuable source for further investigation in HD research. They can be further prioritized based on the calculated property value, that is, the polar surface area (PSA) as being good candidates for passing the Blood-Brain-Barrier (with PSA <60 ^2^) [28]. Table 2 illustrates drug targets associated with the four biological processes as for example, we found Imatinib, associated with the gene ABL1 that targets autophagy and is known to retard production of beta-amyloid [29]. The complete list of the drug targets and also the full drug description can be found at Additional file 1. Figure 6 demonstrates the SPARQL query, separated in different colours that indicate the different data sources that were part of this data integration. Due to the size of the datasets that are involved, this query is not available on the same end-point as the previous queries. Instead we performed this query on a temporary instance of a cloud node running the Virtuoso 7.0 Open Source triple store. We note that no dataset conversions were necessary: RDF datasets were downloaded directly from their respective sources and loaded in our triple store.At this point it is worth mentioning implementation decisions that had to be made. First, we decided to include the GO ontology (rather than the bio2RDF GO data source) because we wanted to make use of the ontology hierarchical structure to obtain all child concepts associated with the GO concepts of interest. This is something that is not readily possible with the bio2RDF data source, because it does not include the GO ontology itself. Secondly, we had to perform a mapping between the GO ontology and the bio2RDF data source because they are using different URIs to represent the same GO term. Figure 5B depicts this part of the query. Although it is possible to execute this as part of the main integration query (Figure 6B), experimentation showed that our particular triple store has better performance when the URI mappings were stored first. In Figure 6A we present the original SPARQL query we created to insert the GO terms and their corresponding subclassses from the GO ontology to the graph stored locally.

**Table 2:** **Drug target results from the data integration query**

Gene	GeneSymbol	GoTerm	Target	Drug	DrugDescription ^[3]^
http://bio2rdf.org/geneid:25	"ABL1"	"autophagy"@en	http://bio2rdf.org/drugbank_target:17	http://bio2rdf.org/drugbank:DB00171	Adenosine triphosphate (ATP)
				http://bio2rdf.org/drugbank:DB00619	Imatinib
				http://bio2rdf.org/drugbank:DB01254	Dasatinib
				http://bio2rdf.org/drugbank:DB04868	Nilotinib
http://bio2rdf.org/geneid:2280	"FKBP1A"	"protein folding"@en	http://bio2rdf.org/drugbank_target:768	http://bio2rdf.org/drugbank:DB00337	Pimecrolimus
				http://bio2rdf.org/drugbank:DB00864	Tacrolimus
				http://bio2rdf.org/drugbank:DB00877	Sirolimus
http://bio2rdf.org/geneid:10105	"PPIF"	"protein folding"@en	http://bio2rdf.org/drugbank_target:2554	http://bio2rdf.org/drugbank:DB00172	L-Proline
http://bio2rdf.org/geneid:5478	"PPIA"		http://bio2rdf.org/drugbank_target:1524		
http://bio2rdf.org/geneid:5479	"PPIB"		http://bio2rdf.org/drugbank_target:4084		
http://bio2rdf.org/geneid:5480	"PPIC"		http://bio2rdf.org/drugbank_target:4085		
http://bio2rdf.org/geneid:7277	"TUBA4A"	"protein folding"@en	http://bio2rdf.org/drugbank_target:2539	http://bio2rdf.org/drugbank:DB00541	Vincristine
				http://bio2rdf.org/drugbank:DB06772	cabazitaxel
				http://bio2rdf.org/drugbank:DB01179	Podofilox
http://bio2rdf.org/geneid:5707	"PSMD1"	proteasomal protein catabolic process	http://bio2rdf.org/drugbank_target:515	http://bio2rdf.org/drugbank:DB00188	Bortezomib

^[3]^Although not drugs in the strict sense, these ligands may serve as starting point for (rational) drug design efforts for these targets.

In the end, the query ran within 15 seconds and retrieved the results as given in Table 2. A detailed discussion of the results is out of the scope of this paper. However, we note that the effort required to integrate these data is relatively minimal: aside from loading the data sources, it approximately takes only an hour to construct the query. Moreover, as is indicated in Figure 6, the query itself is modular: consisting of specific sections related to specific datasets. Therefore extending the query to include other datasets is not very difficult.

## Conclusion

To date there is an enormous amount of valuable information that has been produced by expensive experiments, but remains lost in databases and other repositories that are not easily accessed or processed automatically. This results not only in replicating experiments that have already been performed, but also in preventing all those associations from being tested or reused for building new hypotheses. This paper presents a method that enables life scientists to (i) expose the results from an analysis as scientific assertions, (ii) claim these as their contribution and (iii) provide provenance of the analysis as reference for the claimed assertions. We demonstrated an example from research in Huntington’s Disease. In addition, we presented examples of nanopublication integration in the context of HD, and examples of how nanopublications can facilitate more sophisticated queries, integrating datasets from different research domains. The models for these nanopublications can be used as templates to create similar nanopublications, while the extension to the RO model can also be used to aggregate resources from other experiments that do not involve scientific workflows. Nanopublication provides an incentive for scientists to expose the results from individual experiments and make them available for future exploitation. This ultimately facilitates research across datasets that we anticipate will provide new insights about disease mechanisms. Research can become more efficient and go beyond monolithic journal publication [30].

## Endnotes

^a^ in our case the biological region is: CpG island or one of the chromatin states, active /*weak*/poised promoter or heterochromatic (see Figure 1 and Figure 2).

^b^ All ontologies mentioned in this paper are available through http://bioportal.bioontology.org/ontologies.

## Electronic supplementary material

Additional file 1: Drug target information. This document contains the extended list that resulted from the data integration query concerning drug target information for the four biological processes. (DOC 99 KB)
