# Supplementary material for: Nanopublications for exposing experimental data in the life-sciences: a Huntington’s Disease case study
Source: J Biomed Semantics. 2015 Feb 9;6:5. doi: 10.1186/2041-1480-6-5 (PMC4603842; doi:10.1186/2041-1480-6-5)
Supplement: Supplementary file 1 — Additional file 1: Drug target information. This document contains the extended list that resulted from the data integration query concerning drug target information for the four biological processes. (DOC 99 KB) [file 13326_2014_216_MOESM1_ESM.doc]

**GO:0010498**

| gene | geneSymbol | goTerm | goTermLabel | target | drug | drugDescription |
| --- | --- | --- | --- | --- | --- | --- |
| <http://bio2rdf.org/geneid:5707> | "PSMD1" | [http://purl.org/obo/owl/GO#GO_0031145](http://purl.org/obo/owl/GO#GO_0031145) | "anaphase-promoting complex-dependent proteasomal ubiquitin-dependent protein catabolic process"@en | <http://bio2rdf.org/drugbank_target:515> | <http://bio2rdf.org/drugbank:DB00188> | "Bortezomib is a drug that inhibits the mammalian 26S proteasome. The ubiquitin-proteasome pathway plays an essential role in regulating the intracellular concentration of specific proteins, thereby maintaining homeostasis within cells. Inhibition of the 26S proteasome prevents this targeted proteolysis, which can affect multiple signaling cascades within the cell. This disruption of normal homeostatic mechanisms can lead to cell death. Experiments have demonstrated that bortezomib is cytotoxic to a variety of cancer cell types in vitro. Bortezomib causes a delay in tumor growth in vivo in nonclinical tumor models, including multiple myeloma. Tumor cells, that is, rapidly dividing cells, appear to be more sensitive to proteasome inhibition." |

**GO:0006914**

| gene | geneSymbol | goTerm | goTermLabel | target | drug | drugDescription |
| --- | --- | --- | --- | --- | --- | --- |
| <http://bio2rdf.org/geneid:25> | "ABL1" | [http://purl.org/obo/owl/GO#GO_0006914](http://purl.org/obo/owl/GO#GO_0006914) | "autophagy"@en | <http://bio2rdf.org/drugbank_target:17> | <http://bio2rdf.org/drugbank:DB00171> | "Adenosine triphosphate (ATP) is the nucleotide known in biochemistry as the "molecular currency" of intracellular energy transfer; that is, ATP is able to store and transport chemical energy within cells. ATP also plays an important role in the synthesis of nucleic acids. The total quantity of ATP in the human body is about 0.1 mole. The energy used by human cells requires the hydrolysis of 200 to 300 moles of ATP daily. This means that each ATP molecule is recycled 2000 to 3000 times during a single day. ATP cannot be stored, hence its consumption must closely follow its synthesis." |
| <http://bio2rdf.org/geneid:25> | "ABL1" | [http://purl.org/obo/owl/GO#GO_0006914](http://purl.org/obo/owl/GO#GO_0006914) | "autophagy"@en | <http://bio2rdf.org/drugbank_target:17> | <http://bio2rdf.org/drugbank:DB00619> | "Imatinib is an antineoplastic agent used to treat chronic myelogenous leukemia. Imatinib is a 2-phenylaminopyrimidine derivative that functions as a specific inhibitor of a number of tyrosine kinase enzymes. In chronic myelogenous leukemia, the Philadelphia chromosome leads to a fusion protein of Abl with Bcr (breakpoint cluster region), termed Bcr-Abl. As this is now a continuously active tyrosine kinase, Imatinib is used to decrease Bcr-Abl activity." |
| <http://bio2rdf.org/geneid:25> | "ABL1" | [http://purl.org/obo/owl/GO#GO_0006914](http://purl.org/obo/owl/GO#GO_0006914) | "autophagy"@en | <http://bio2rdf.org/drugbank_target:17> | <http://bio2rdf.org/drugbank:DB01254> | "Dasatinib is an oral dual BCR/ABL and Src family tyrosine kinase inhibitor" |
| <http://bio2rdf.org/geneid:25> | "ABL1" | [http://purl.org/obo/owl/GO#GO_0006914](http://purl.org/obo/owl/GO#GO_0006914) | "autophagy"@en | <http://bio2rdf.org/drugbank_target:17> | <http://bio2rdf.org/drugbank:DB04868> | "Nilotinib is a transduction inhibitor that targets BCR-ABL, c-kit and PDGF, for the potential treatment of various leukemias, including chronic myeloid leukemia (CML)." |

**GO:0006457**

| gene | geneSymbol | goTerm | goTermLabel | target | drug | drugDescription |
| --- | --- | --- | --- | --- | --- | --- |
| <http://bio2rdf.org/geneid:2280> | "FKBP1A" | [http://purl.org/obo/owl/GO#GO_0006458](http://purl.org/obo/owl/GO#GO_0006458) | "'de novo' protein folding"@en | <http://bio2rdf.org/drugbank_target:768> | <http://bio2rdf.org/drugbank:DB00337> | "Pimecrolimus is a chemical that is used to treat atopic dermatitis (eczema). Atopic dermatitis is a skin condition characterized by redness, itching, scaling and inflammation of the skin. The cause of atopic dermatitis is not known; however, scientists believe that it may be due to activation of the immune system by various environmental or emotional triggers. Scientists do not know exactly how pimecrolimus reduces the manifestations of atopic dermatitis, but pimecrolimus reduces the action of T-cells and mast cells which are part of the immune system and contribute to responses of the immune system. Pimecrolimus prevents the activation of T-cells by blocking the effects of chemicals (cytokines) released by the body that stimulate T-cells. Pimecrolimus also reduces the ability of mast cells to release chemicals that promote inflammation." |
| <http://bio2rdf.org/geneid:2280> | "FKBP1A" | [http://purl.org/obo/owl/GO#GO_0006458](http://purl.org/obo/owl/GO#GO_0006458) | "'de novo' protein folding"@en | <http://bio2rdf.org/drugbank_target:768> | <http://bio2rdf.org/drugbank:DB00864> | "Tacrolimus is a macrolide antibiotic. It acts by reducing peptidyl-prolyl isomerase activity by binding to the immunophilin FKBP-12 (FK506 binding protein) creating a new complex. This inhibits both T-lymphocyte signal transduction and IL-2 transcription. Although this activity is similar to cyclosporine studies have shown that the incidence of acute rejection is reduced by tacrolimus use over cyclosporine. Tacrolimus has also been shown to be effective in the topical treatment of eczema, particularly atopic eczema. It suppresses inflammation in a similar way to steroids, but is not as powerful. An important dermatological advantage of tacrolimus is that it can be used directly on the face; topical steroids cannot be used on the face, as they thin the skin dramatically there. On other parts of the body, topical steroid are generally a better treatment." |
| <http://bio2rdf.org/geneid:2280> | "FKBP1A" | [http://purl.org/obo/owl/GO#GO_0006458](http://purl.org/obo/owl/GO#GO_0006458) | "'de novo' protein folding"@en | <http://bio2rdf.org/drugbank_target:768> | <http://bio2rdf.org/drugbank:DB00877> | "Sirolimus, a macrocyclic lactone produced by <i>Streptomyces hygroscopicus</i>, is an immunosuppressive agent indicated for the prophylaxis of organ rejection in patients receiving renal transplants. It is recommended that sirolimus be used in a regimen with cyclosporine and corticosteroids." |
| <http://bio2rdf.org/geneid:2280> | "FKBP1A" | [http://purl.org/obo/owl/GO#GO_0006457](http://purl.org/obo/owl/GO#GO_0006457) | "protein folding"@en | <http://bio2rdf.org/drugbank_target:768> | <http://bio2rdf.org/drugbank:DB00337> | "Pimecrolimus is a chemical that is used to treat atopic dermatitis (eczema). Atopic dermatitis is a skin condition characterized by redness, itching, scaling and inflammation of the skin. The cause of atopic dermatitis is not known; however, scientists believe that it may be due to activation of the immune system by various environmental or emotional triggers. Scientists do not know exactly how pimecrolimus reduces the manifestations of atopic dermatitis, but pimecrolimus reduces the action of T-cells and mast cells which are part of the immune system and contribute to responses of the immune system. Pimecrolimus prevents the activation of T-cells by blocking the effects of chemicals (cytokines) released by the body that stimulate T-cells. Pimecrolimus also reduces the ability of mast cells to release chemicals that promote inflammation." |
| <http://bio2rdf.org/geneid:2280> | "FKBP1A" | [http://purl.org/obo/owl/GO#GO_0006457](http://purl.org/obo/owl/GO#GO_0006457) | "protein folding"@en | <http://bio2rdf.org/drugbank_target:768> | <http://bio2rdf.org/drugbank:DB00864> | "Tacrolimus is a macrolide antibiotic. It acts by reducing peptidyl-prolyl isomerase activity by binding to the immunophilin FKBP-12 (FK506 binding protein) creating a new complex. This inhibits both T-lymphocyte signal transduction and IL-2 transcription. Although this activity is similar to cyclosporine studies have shown that the incidence of acute rejection is reduced by tacrolimus use over cyclosporine. Tacrolimus has also been shown to be effective in the topical treatment of eczema, particularly atopic eczema. It suppresses inflammation in a similar way to steroids, but is not as powerful. An important dermatological advantage of tacrolimus is that it can be used directly on the face; topical steroids cannot be used on the face, as they thin the skin dramatically there. On other parts of the body, topical steroid are generally a better treatment." |
| <http://bio2rdf.org/geneid:2280> | "FKBP1A" | [http://purl.org/obo/owl/GO#GO_0006457](http://purl.org/obo/owl/GO#GO_0006457) | "protein folding"@en | <http://bio2rdf.org/drugbank_target:768> | <http://bio2rdf.org/drugbank:DB00877> | "Sirolimus, a macrocyclic lactone produced by <i>Streptomyces hygroscopicus</i>, is an immunosuppressive agent indicated for the prophylaxis of organ rejection in patients receiving renal transplants. It is recommended that sirolimus be used in a regimen with cyclosporine and corticosteroids." |
| <http://bio2rdf.org/geneid:2280> | "FKBP1A" | [http://purl.org/obo/owl/GO#GO_0042026](http://purl.org/obo/owl/GO#GO_0042026) | "protein refolding"@en | <http://bio2rdf.org/drugbank_target:768> | <http://bio2rdf.org/drugbank:DB00337> | "Pimecrolimus is a chemical that is used to treat atopic dermatitis (eczema). Atopic dermatitis is a skin condition characterized by redness, itching, scaling and inflammation of the skin. The cause of atopic dermatitis is not known; however, scientists believe that it may be due to activation of the immune system by various environmental or emotional triggers. Scientists do not know exactly how pimecrolimus reduces the manifestations of atopic dermatitis, but pimecrolimus reduces the action of T-cells and mast cells which are part of the immune system and contribute to responses of the immune system. Pimecrolimus prevents the activation of T-cells by blocking the effects of chemicals (cytokines) released by the body that stimulate T-cells. Pimecrolimus also reduces the ability of mast cells to release chemicals that promote inflammation." |
| <http://bio2rdf.org/geneid:2280> | "FKBP1A" | [http://purl.org/obo/owl/GO#GO_0042026](http://purl.org/obo/owl/GO#GO_0042026) | "protein refolding"@en | <http://bio2rdf.org/drugbank_target:768> | <http://bio2rdf.org/drugbank:DB00864> | "Tacrolimus is a macrolide antibiotic. It acts by reducing peptidyl-prolyl isomerase activity by binding to the immunophilin FKBP-12 (FK506 binding protein) creating a new complex. This inhibits both T-lymphocyte signal transduction and IL-2 transcription. Although this activity is similar to cyclosporine studies have shown that the incidence of acute rejection is reduced by tacrolimus use over cyclosporine. Tacrolimus has also been shown to be effective in the topical treatment of eczema, particularly atopic eczema. It suppresses inflammation in a similar way to steroids, but is not as powerful. An important dermatological advantage of tacrolimus is that it can be used directly on the face; topical steroids cannot be used on the face, as they thin the skin dramatically there. On other parts of the body, topical steroid are generally a better treatment." |
| <http://bio2rdf.org/geneid:2280> | "FKBP1A" | [http://purl.org/obo/owl/GO#GO_0042026](http://purl.org/obo/owl/GO#GO_0042026) | "protein refolding"@en | <http://bio2rdf.org/drugbank_target:768> | <http://bio2rdf.org/drugbank:DB00877> | "Sirolimus, a macrocyclic lactone produced by <i>Streptomyces hygroscopicus</i>, is an immunosuppressive agent indicated for the prophylaxis of organ rejection in patients receiving renal transplants. It is recommended that sirolimus be used in a regimen with cyclosporine and corticosteroids." |
| <http://bio2rdf.org/geneid:2280> | "FKBP1A" | [http://purl.org/obo/owl/GO#GO_0022417](http://purl.org/obo/owl/GO#GO_0022417) | "protein maturation by protein folding"@en | <http://bio2rdf.org/drugbank_target:768> | <http://bio2rdf.org/drugbank:DB00337> | "Pimecrolimus is a chemical that is used to treat atopic dermatitis (eczema). Atopic dermatitis is a skin condition characterized by redness, itching, scaling and inflammation of the skin. The cause of atopic dermatitis is not known; however, scientists believe that it may be due to activation of the immune system by various environmental or emotional triggers. Scientists do not know exactly how pimecrolimus reduces the manifestations of atopic dermatitis, but pimecrolimus reduces the action of T-cells and mast cells which are part of the immune system and contribute to responses of the immune system. Pimecrolimus prevents the activation of T-cells by blocking the effects of chemicals (cytokines) released by the body that stimulate T-cells. Pimecrolimus also reduces the ability of mast cells to release chemicals that promote inflammation." |
| <http://bio2rdf.org/geneid:2280> | "FKBP1A" | [http://purl.org/obo/owl/GO#GO_0022417](http://purl.org/obo/owl/GO#GO_0022417) | "protein maturation by protein folding"@en | <http://bio2rdf.org/drugbank_target:768> | <http://bio2rdf.org/drugbank:DB00864> | "Tacrolimus is a macrolide antibiotic. It acts by reducing peptidyl-prolyl isomerase activity by binding to the immunophilin FKBP-12 (FK506 binding protein) creating a new complex. This inhibits both T-lymphocyte signal transduction and IL-2 transcription. Although this activity is similar to cyclosporine studies have shown that the incidence of acute rejection is reduced by tacrolimus use over cyclosporine. Tacrolimus has also been shown to be effective in the topical treatment of eczema, particularly atopic eczema. It suppresses inflammation in a similar way to steroids, but is not as powerful. An important dermatological advantage of tacrolimus is that it can be used directly on the face; topical steroids cannot be used on the face, as they thin the skin dramatically there. On other parts of the body, topical steroid are generally a better treatment." |
| <http://bio2rdf.org/geneid:2280> | "FKBP1A" | [http://purl.org/obo/owl/GO#GO_0022417](http://purl.org/obo/owl/GO#GO_0022417) | "protein maturation by protein folding"@en | <http://bio2rdf.org/drugbank_target:768> | <http://bio2rdf.org/drugbank:DB00877> | "Sirolimus, a macrocyclic lactone produced by <i>Streptomyces hygroscopicus</i>, is an immunosuppressive agent indicated for the prophylaxis of organ rejection in patients receiving renal transplants. It is recommended that sirolimus be used in a regimen with cyclosporine and corticosteroids." |
| <http://bio2rdf.org/geneid:10105> | "PPIF" | [http://purl.org/obo/owl/GO#GO_0006457](http://purl.org/obo/owl/GO#GO_0006457) | "protein folding"@en | <http://bio2rdf.org/drugbank_target:2554> | <http://bio2rdf.org/drugbank:DB00172> | "L-Proline is a major amino acid found in cartilage and is important for maintaining youthful skin as well as repair of muscle, connective tissue and skin damage. It is also essential for the immune system, and for necessary balance of this formula. It is an essential component of collagen and is important for proper functioning of joints and tendons. L-Proline is extremely important for the proper functioning of joints and tendons. Helps maintain and strengthen heart muscles." |
| <http://bio2rdf.org/geneid:5478> | "PPIA" | [http://purl.org/obo/owl/GO#GO_0006457](http://purl.org/obo/owl/GO#GO_0006457) | "protein folding"@en | <http://bio2rdf.org/drugbank_target:1524> | <http://bio2rdf.org/drugbank:DB00172> | "L-Proline is a major amino acid found in cartilage and is important for maintaining youthful skin as well as repair of muscle, connective tissue and skin damage. It is also essential for the immune system, and for necessary balance of this formula. It is an essential component of collagen and is important for proper functioning of joints and tendons. L-Proline is extremely important for the proper functioning of joints and tendons. Helps maintain and strengthen heart muscles." |
| <http://bio2rdf.org/geneid:5479> | "PPIB" | [http://purl.org/obo/owl/GO#GO_0006457](http://purl.org/obo/owl/GO#GO_0006457) | "protein folding"@en | <http://bio2rdf.org/drugbank_target:4084> | <http://bio2rdf.org/drugbank:DB00172> | "L-Proline is a major amino acid found in cartilage and is important for maintaining youthful skin as well as repair of muscle, connective tissue and skin damage. It is also essential for the immune system, and for necessary balance of this formula. It is an essential component of collagen and is important for proper functioning of joints and tendons. L-Proline is extremely important for the proper functioning of joints and tendons. Helps maintain and strengthen heart muscles." |
| <http://bio2rdf.org/geneid:5480> | "PPIC" | [http://purl.org/obo/owl/GO#GO_0006457](http://purl.org/obo/owl/GO#GO_0006457) | "protein folding"@en | <http://bio2rdf.org/drugbank_target:4085> | <http://bio2rdf.org/drugbank:DB00172> | "L-Proline is a major amino acid found in cartilage and is important for maintaining youthful skin as well as repair of muscle, connective tissue and skin damage. It is also essential for the immune system, and for necessary balance of this formula. It is an essential component of collagen and is important for proper functioning of joints and tendons. L-Proline is extremely important for the proper functioning of joints and tendons. Helps maintain and strengthen heart muscles." |
| <http://bio2rdf.org/geneid:7277> | "TUBA4A" | [http://purl.org/obo/owl/GO#GO_0006457](http://purl.org/obo/owl/GO#GO_0006457) | "protein folding"@en | <http://bio2rdf.org/drugbank_target:2539> | <http://bio2rdf.org/drugbank:DB00541> | "Vincristine is a vinca alkaloid antineoplastic agent used as a treatment for various cancers including breast cancer, Hodgkin's disease, Kaposi's sarcoma, and testicular cancer. The vinca alkaloids are structurally similar compounds comprised of 2 multiringed units, vindoline and catharanthine. The vinca alkaloids have become clinically useful since the discovery of their antitumour properties in 1959. Initially, extracts of the periwinkle plant (Catharanthus roseus) were investigated because of putative hypoglycemic properties, but were noted to cause marrow suppression in rats and antileukemic effects <i>in vitro</i>. Vincristine binds to the microtubular proteins of the mitotic spindle, leading to crystallization of the microtubule and mitotic arrest or cell death. Vincristine has some immunosuppressant effect. The vinca alkaloids are considered to be cell cycle phase-specific." |
| <http://bio2rdf.org/geneid:7277> | "TUBA4A" | [http://purl.org/obo/owl/GO#GO_0006457](http://purl.org/obo/owl/GO#GO_0006457) | "protein folding"@en | <http://bio2rdf.org/drugbank_target:2539> | <http://bio2rdf.org/drugbank:DB06772> | "After an intravenous dose of cabazitaxel 25 mg/m2 every three weeks to a population of 170 patients with solid tumors, the mean Cmax in patients with metastatic prostate cancer was 226 ng/mL (CV 107%) and was reached at the end of the one-hour infusion (Tmax). The mean AUC in patients with metastatic prostate cancer was 991 ng.h/mL (CV 34%)." |
| <http://bio2rdf.org/geneid:7277> | "TUBA4A" | [http://purl.org/obo/owl/GO#GO_0006457](http://purl.org/obo/owl/GO#GO_0006457) | "protein folding"@en | <http://bio2rdf.org/drugbank_target:2539> | <http://bio2rdf.org/drugbank:DB01179> | "Podofilox, also called podophyllotoxin, is a purer and more stable form of podophyllin in which only the biologically active portion of the compound is present. Podofilox is used to remove certain types of warts on the outside skin of the genital areas." |

Note: In these tables the items are labeled as drugs but not in the strict sense, these ligands may serve as starting point for (rational) drug design efforts for these targets
